# Energy balance and macronutrient distribution in relation to C-reactive protein and HbA1c levels among patients with type 2 diabetes

**DOI:** 10.3402/fnr.v60.29904

**Published:** 2016-05-27

**Authors:** Hiba Bawadi, Rami Katkhouda, Ahmad Al-Haifi, Reema Tayyem, Cosette Fakih Elkhoury, Zeina Jamal

**Affiliations:** 1 College of Health Sciences, Qatar University, Doha, Qatar; 2 Department of Nutrition and Food Technology, Jordan University of Science and Technology, Irbid, Jordan; 3 Food and Nutrition Science, College of Health Sciences, Showaikh, Kuwait; 4 Department of Nutrition and Food Technology, Faculty of Agriculture, The University of Jordan, Amman, Jordan

**Keywords:** type 2 diabetes, hs-CRP, HbA1c, energy balance

## Abstract

**Background:**

Recently growing evidence indicates that obesity and diabetes are states of inflammation associated with elevated circulation of inflammatory mediators. Excess adiposity and oxidative stress, induced by feeding, may also lead to a state of low-grade inflammation.

**Objective:**

This study aimed at investigating energy balance and distribution in relation to low-grade inflammation among patients with type 2 diabetes.

**Design:**

A cross-sectional study included 198 male and female patients with type 2 diabetes. Patients’ weight, height, waist circumference, total body fat and truncal fat percent, energy, and macronutrient intake were measured. Venous blood specimens were collected, and levels of HbA1c and serum levels of high-sensitivity C-reactive protein (hs-CRP) were determined.

**Results:**

After adjusting for covariates (body mass index, total body fat, and truncal fat), energy balance was positively correlated with hs-CRP and HbA1c. A positive energy balance was also associated with increased waist circumference and truncal fat percent (*p*<0.05). Total energy intake, percent energy from fat (*p*=0.04), and percent energy from proteins (*p*=0.03), but not percent energy from carbohydrates (*p*=0.12), were also correlated with higher hs-CRP levels among poorly glycemic-controlled patients.

**Conclusion:**

Positive energy balance is associated with elevations in hs-CRP. Increased energy intake and increased percentages of energy from fat and protein are associated with elevated hs-CRP among patients with poor glycemic control.

Diabetes is among the top four leading causes of death in Jordan (1). It is also considered as one of the most costly chronic diseases with strong association with many chronic diseases, such as renal failure, atherosclerotic vascular disease, hypertension, and dyslipidemia (2). Attaining and sustaining a healthy body weight is a key factor for preventing diabetes and its comorbidities (3). Several studies suggest that accumulated adipose tissues, especially in the abdominal region, are associated with increased insulin resistance, elevated HbA1c levels, and increased diabetes complications (4).

Achieving a balance between energy input and output is crucial in maintaining healthy weight and protecting oneself from chronic diseases. However, achieving this balance seems challenging with high percentage of people shifting this balance toward expending less and/or consuming more of their daily energy intake. Hence, weight gain and obesity are becoming global epidemics. In addition to obesity, excessive caloric consumption is now linked to other chronic diseases, such as heart disease, cancer, and type 2 diabetes mellitus (5). Excessive energy intake is interrelated with low-grade inflammation mediated by increased oxidative stress, abdominal obesity, insulin resistance, and altered metabolism of glucose and fat (6–8).

Low-grade inflammation is a chronic inflammatory response that can promote tissue damage (9). Low-grade inflammation is a hallmark of the progression of type 2 diabetes; it plays a vital role in diabetes pathogenesis through the induction of beta cell apoptosis (10). Several studies investigated the relation between diabetes and inflammation (10, 11); however, this relation was not addressed in depth with regard to energy balance and macronutrients’ contribution to total energy intake. This study aimed to investigate the impact of energy balance and macronutrient distribution on high-sensitivity C-reactive protein (hs-CRP) levels among diabetic patients with good versus poor glycemic control.

## Experimental section

### Participants

The study protocol and questionnaires were approved by the research ethics committee: Institutional Review Board at Jordan University of Science and Technology. Patients were recruited from the outpatient endocrinology unit at King Abdullah University Hospital (KAUH), Jordan University of Science and Technology Health Center, and major private endocrinology clinics in North of Jordan.

Initial screening included 1,500 patients diagnosed with type 2 diabetes. Because of the presence of multiple potential cofounding variables associated with the increased levels of hs-CRP, several exclusion criteria, listed below, were set. Thus, only 13% of the initially screened patients were eligible to complete the study. Patients excluded from the study were those with the following criteria: 1) diagnosed with type 2 diabetes for less than 1 year; 2) diagnosed with rheumatoid arthritis, cancer, diabetic foot, kidney diseases, or any chronic or acute inflammatory disease; 3) had recent major or minor surgery; 4) patients on nonsteroidal anti-inflammatory drugs for less than 2 weeks before the study blood sampling event; 5) women on oral contraceptives; and 6) women who were pregnant. Participants were informed about the objectives and the protocol of the study, and thereafter, they were asked to sign a consent form.

### Blood specimen collection and analysis

A 10-ml sample of venous blood was collected from each patient by a registered nurse. The blood samples were collected in ethylenediaminetetraacetic acid tubes, and HbA1c was measured in whole blood using the immuno-inhibition test for the quantitative determination of glycosylated hemoglobin (Beckman Coulter AU analyzers). Blood samples were collected in Z-Clot activator tubes and allowed to clot before centrifugation for 15 min. Aliquots of serum were stored at ≤−22°C in sterile small tubes before biochemical assay. Immuno-turbidimetric test was used to determine hs-CRP levels (Beckman Coulter AU analyzers).

### Anthropometrics and body composition

Anthropometrics (weight, height, and waist circumference [WC]) were measured according to World Health Organization (WHO) procedures (12). Body weight was measured with the individuals wearing no shoes and light clothing. Height was measured using a measuring rod (Seca, Germany). Body mass index (BMI) was calculated using the ratio of weight (kilograms) to the square of height (meters). WC was measured to the nearest centimeter using nonstretchable circumference measuring tape (SECA 203, Germany). The site of tape placing was determined according to WHO description of middle way between the iliac crest and lower rib border. The BMI cut-off points set by the WHO were used to classify patients (12).

Patients’ total body fat and truncal fat percent were determined using bioelectrical impedance technique (TANITA, BC-418). The segmental body composition analyzer (TANITA, BC-418) used in this study was previously validated against hydrodensitometry in the assessment of body composition in healthy young adults (13).

Body fat and percentage cut-off points used were gender and age specific based on which patients were classified into healthy, overfat, and obese (14). Cut-off points for truncal fat percentage were gender specific, according to which patients were classified into three levels of truncal fat: low, average, and high (14).

### Energy balance and macronutrient distribution

Energy balance was defined when daily consumed energy was equal to energy needs. Hence, a positive energy balance was obtained when energy consumption exceeded the needs, and a negative energy balance was obtained when energy consumption was less than the needs.

Patients’ daily energy intake was assessed using semiquantitative food frequency questionnaire (FFQ). The FFQ was administered by an interview performed by s study was previously validated for use in Jordanian setting (15). Participants were asked about their intake of different food items (109 items were included) during the last year. A 1-year period was selected to count for seasonal variation. Participants were asked how frequently, on average, during the past year they consumed one standard serving of a specific food item in nine categories (<1/month, 2–3/month, 1–2/week, 3–4/week, 5–6/week, 1/day, 2–3/day, 4–5/day, or 6/day). For the purpose of accuracy in portion size estimation, food models and standard measuring tools were used. Responses on the frequency of consumption of a specified serving size for each food item were converted into average daily intake. Dietary intakes were analyzed using a dietary analysis software (ESHA Food Processor SQL version 10.1.1; ESHA, Salem, Oregon). Foods consumed in Jordan and not available in the software were added manually to the database (16).

Patients’ basal metabolic rates (BMRs) were estimated using Mifflin St. Jeor equation as recommended by the US Academy of Nutrition and Dietetics (17). To obtain the patients’ energy needs, estimated BMR was multiplied with the patients’ physical activity-level factor. The physical activity level was determined using a validated international physical activity questionnaire (IPAQ) (18). IPAQ is a standardized measure to estimate habitual practice of physical activity of populations from different cultural and socioeconomic backgrounds. The questionnaire is a 7-day recall of physical activity and includes eight items to estimate the time spent performing physical activity. IPAQ classifies subjects into three categories; low physical activity, moderate physical activity, and high or vigorous physical activity (18).

### Statistical analysis

The Statistical Package for Social Sciences software (SPSS, version 19; SPSS Inc., Chicago, Massachusetts) was used for data processing and data analysis. Descriptive analysis was performed to obtain frequencies, means, and standard deviations. A *p*-value of <0.05 was considered the cut-off level for statistical significance. Total energy and energy from fat, carbohydrates, and proteins were grouped into quartiles. Analysis of covariance was conducted to examine the impact of energy balance and macronutrient distribution on hs-CRP levels and HbA1c levels. Covariates accounted for in the model were age, gender, lipid-lowering drugs, BMI, WC, truncal fat percent, and diabetes duration.

## Results

The study sample comprised mostly females (63.1%) and adults aged between 50 and 80 years with mean age (standard deviation) of 55.9 (9.3). Only one-quarter of the participants received more than 12 years of formal education. Almost half of the participants were recently diagnosed with diabetes (<5 years). The means of BMI and HbA1c of the study sample were 32.8 kg/m^2^ and 8.1%, respectively (Table 1). Study variables were presented according to patients’ glycemic control. As Table 1 shows, patients with poor glycemic control had higher BMI, body fat percentage, truncal fat percentage, WC, CRP, and caloric intake as compared with patients with good glycemic control.

**Table 1:** Socio-demographic and relevant characteristics of the participants according to glycemic control

Variable	Good glycemic control (*n*=74)	Poor glycemic control (*n*=124)	Total (*n*=198)
Gender (*n* [%])			
Male	26 (35.1)	47 (37.9)	73 (36.9)
Female	48 (64.9)	77 (62.1)	125 (63.1)
Diabetes duration (*n* [%], years)			
<5	50 (67.6)	44 (36.1)	94 (47.4)
6–12	13 (17.6)	48 (39.3)	61 (30.8)
13–19	8 (10.8)	16 (13.1)	24 (12.1)
>20	3 (4.1)	14 (11.5)	17 (8.6)
Years treated with insulin (*n* [%])	6 (8.1)	34 (27.4)	40 (20.2)
Years of formal education (*n* [%])			
Illiterate	10 (14.3)	19 (15.7)	29 (14.6)
≤12 years	41 (58.6)	70 (57.9)	118 (59.6)
>12 years	19 (27.1)	32 (26.4)	51 (25.8)
Mean (±SD) age (years)	56.1±10.3	55.7±8.7	55.9±9.3
Mean (±SD) body mass index (kg/m^2^)	31.8±5.1^a^	33.2±5.7^b^	32.8±5.9
Mean (±SD) % body fat	35.6±9.6^a^	36.5±9.5^b^	36.1±9.6
Mean (±SD) % truncal fat	33.5±5.2^a^	34.3±8.6^b^	34.0±8.5
Mean (±SD) WC (cm)	101.2±11.1^a^	107.0±12.3^b^	105.0±12.2
Mean (±SD) hs-CRP (mg/L)	7.4±9.9^a^	9.6±9.1^b^	8.8±9.5
Mean (±SD) total daily energy (kcal)	2043±820.7^a^	2416.2±987.4^b^	2279.2±944.4
Mean (±SD) % of energy from carbohydrates	55.7±8.2	57.3±7.1	56.7±7.5
Mean (±SD) % of energy from fat	28.5±6.7	27.1±6.3	27.7±6.5
Mean (±SD) % of energy from protein	15.7±3.3	15.7±3.0	15.9±3.1

Superscripts were based on post hoc mean differences analysis. Means with similar superscripts are not statistically different. hs-CRP=high-sensitivity C-reactive protein; SD=standard deviation.

WC and truncal fat percentage were significantly higher among subjects with positive energy balance (*p*<0.05). It is worth noticing that there was a trend of association between BMI and positive energy balance. However, it was not of statistical significance (*p*=0.057) (Table 2). Participants with a positive energy balance had significantly higher mean values for hs-CRP and HbA1c levels compared with those with negative energy balance (Table 3).

**Table 2:** Anthropometric measurements of patients with positive versus negative energybalance (*N* = 198)

Energy balance^a^	BMI	WC	% Body fat	% Truncal fat
Negative (*n*=82)	32.8±5.5	104.2±11.1	35.1±8.9	34.2±8.1
Positive (*n*=116)	33.2±6.4	106.5±13.3	37.6±8.4	34.6±8.1
*p*	0.057	0.012	0.050	0.002

All values are mean±standard error of the mean. *P*-values were obtained after adjustment for age, income, and treatment with insulin.

BMI=body mass index in kg/m^2^; WC=waist circumference in cm.

a Energy balance=energy intake – energy expended.

**Table 3:** Levels of hs-CRP and HbA1c of patients with positive versus negative energybalance (*N* = 198)

Energy balance^a^	hs-CRP (mg/L)	HbA1c (%)
Negative (*n*=82)	6.7±4.7	7.8±1.7
Positive (*n*=116)	9.2±6.2	8.41±2.0
*p*	0.008	0.008

All values are mean±standard error of the mean. *P*-values were determined after adjustment for BMI, truncal fat, and total body fat. hs-CRP=high-sensitivity C-reactive protein.

a Energy balance=energy intake – energy expended.

Table 4 shows the association between participants’ hs-CRP levels in relation to their daily total energy intake and macronutrients’ contribution to energy intake. Participants were grouped into good and poor glycemic control for comparison purposes, and macronutrient and energy consumptions were divided into quartiles. Total energy, energy from protein, and energy from fat were significantly associated with higher hs-CRP serum levels among participants with poor glycemic control at the highest quartile of consumption (*p*<0.05). We found no statistically significant relationship with carbohydrate consumptions across all quartiles.

**Table 4:** Levels of hs-CRP across different quartiles of energy intakes and distribution among patients with good or poor glycemic control

	Good glycemic control (*n* = 67)					Poor glycemic control (*n* = 131)				
				
Quartiles	1	2	3	4	*P*	1	2	3	4	*P*
Total energy intake	6.2±0.9^a^	8.6±4.6^a^	5.8±1.6^a^	9.6±1.8^a^	0.287	8.5±1.3^ab^	8.4±1.2^a^	8.2±1.0^a^	12.6±2.4^b^	0.044
% energy from carbohydrates	6.3±0.9^a^	11.1±5.7^a^	5.7±1.3^a^	8.1±1.7^a^	0.341	10.0±1.7^a^	8.0±0.9^a^	7.9±1.0^a^	12.1±2.3^a^	0.120
% energy from proteins	8.3±3.2^a^	5.0±1.1^a^	7.5±1.6^a^	8.8±2.1^a^	0.720	7.3±1.2^a^	9.7±1.2^ab^	8.00±1.0^a^	12.2±2.4^b^	0.030
% energy from fats	4.3±0.8^a^	5.8±1.0^a^	11.3±5.2^a^	10.2±1.9^a^	0.114	7.2±1.1^a^	8.2±1.0^a^	9.5±1.2^ab^	12.4±2.4^b^	0.044

All values are mean±standard error of the mean. *P*-values were determined after adjustment for age, gender, lipid-lowering drugs, and diabetes duration. Good glycemic control was defined as HbA1c <7%, according to ADA (2009).

Superscripts were based on post hoc mean differences analysis. Means with similar superscripts are not statistically different. hs-CRP = high-sensitivity C-reactive protein.

## Discussion

The current study showed that positive energy balance was associated with increased levels of hs-CRP among patients with poorly controlled diabetes.

There is a strong relationship between the low-grade inflammation that results from ‘lipotoxicity’ and the increased expression of inflammatory markers. This state causes changes in responses and signaling of adipocytes, which in turn has shown to interfere with normal insulin signaling (19).

Generally, inflammation is a healthy physiological reaction to a harmful stimuli and such response is aimed at restoring homeostasis (20); however, although it is strange to apply this definition to feeding, it seems that an excessive energy consumption induces a low-grade inflammation similar to that triggered by harmful stimuli. Unfortunately, this inflammatory status does not restore homeostasis, but disrupts glucose metabolism (20).

In the current study, the effect of positive energy balance on hs-CRP and HbA1c was investigated. Higher levels of hs-CRP and HbA1c among patients with positive energy balance were reported. Such results were observed even after adjusting for BMI and body composition. Our findings confirm the role of excess feeding on inflammation and glycemic control (20). We also found a significant association between a positive energy balance and WC, percentage of fat, and truncal fat. This further adds to the theory that a positive energy balance may cause metabolic impairments yielding low-grade inflammation and impaired glucose metabolism.

Our results are promising if weight loss regimens, inducing either a negative energy balance or a decreased fat mass, can reverse this low-grade inflammation. This may mean that simply creating a negative energy balance through dietary manipulations would decrease the inflammatory state, which impacts on the development and progression of diabetes and its complications. Petelin et al. performed a clinical trial that induced weight loss by negative energy balance and were able to show a lowered inflammation and decreased insulin levels among the participants (21).

Interestingly, our findings showed not only an effect on inflammation from positive energy balance but also a specific effect of dietary fats and proteins. We grouped the macronutrient intake into quartiles across subjects with good versus poor glycemic control; we found higher hs-CRP values among subjects with poor glycemic control and with highest quartiles of dietary fats and proteins consumption (Table 4).

In accordance with our study, Baer et al. also found hat a high-fat diet significantly increased circulatory concentrations of CRP (22). Such results may be attributed to the high potential of fat to initiate oxidative stress (23), possibly leading to systemic inflammation (24). Despite the fact that current research did not investigate the relation between trans-fat and inflammation, the literature supports the relation between trans-fat and chronic inflammation. In fact, Mozaffarian et al. found a positive association between trans-fat intakes in women and elevation of inflammatory markers (25).

We also found an association between dietary proteins and inflammation. This relation may be attributed to end products of processing protein-rich foods, which may be the source of oxidation. According to Uribarri et al., dietary advanced glycation end products are produced in high heat processing of protein-rich foods and are associated with oxidative stress (26). Carbohydrates, on the other hand, are quite controversial in their role in low-grade inflammation. In fact, it is often the high glycemic index of a food that is associated with increased inflammatory markers rather than the amounts of carbohydrates. Kallio et al. found that long-term consumption of different cereals has different effects on postprandial insulin secretions, which may be modulating the inflammatory process (27). According to the authors, a diet rich in whole grains tends to have a noninflammatory effect, whereas a diet high in glycemic index holds the culprit in inflammation. We did not find a relationship between carbohydrate intake and inflammation in our study, indicating a more protective role of carbohydrates as compared with fats and proteins.

It is, however, essential to mention that we have found the associations with macronutrients to be statistically significant only among the subjects with poor glycemic control at the highest quartiles of energy intake, indicating that dietary interventions aiming to prevent overconsumption of energy would specifically be of value for patients with poor glycemic control.

The findings of this study may be limited because of several factors. The cross-sectional nature of this study did not allow us to investigate the causal relationship between energy intake and macronutrient distribution with inflammation. The lengthy questionnaire and extended patient interview time may have resulted in respondents’ fatigue, which may impact the accuracy of their responses. A major limitation of this study is that energy expenditure was estimated using predictive equations. The use of indirect calorimetry might have provided more accurate information than using estimation equations.

## Conclusions

In conclusion, positive energy balance might be associated with elevated hs-CRP among patients with poorly controlled diabetes. Dietary fats and proteins seemed to be associated with higher hs-CRP levels among subjects with poor glycemic control. Our findings indicate that energy balance may play a role in the low-grade inflammatory processes underlying obesity-related conditions and particularly diabetes.
